# Most Elite Athletes Who Underwent Hip Arthroscopy for Femoroacetabular Impingement Syndrome Did Not Return to the Same Level of Sport, but the Majority Were Satisfied With the Outcome of Surgery

**DOI:** 10.1016/j.asmr.2021.12.021

**Published:** 2022-02-13

**Authors:** Thorkell Snaebjörnsson, Sofie Sjövall Anari, Ida Lindman, Neel Desai, Anders Stålman, Olufemi R. Ayeni, Axel Öhlin

**Affiliations:** a Department of Orthopaedics, Institute of Clinical Sciences, Sahlgrenska Academy, Gothenburg University, Gothenburg, Sweden; b Department of Orthopaedics, Sahlgrenska University Hospital, Mölndal, Sweden; c Capio Artro Clinic, FIFA Medical Centre of Excellence, Sophiahemmet Hospital, Stockholm, Sweden; d Department of Molecular Medicine and Surgery, Stockholm Sports Trauma Research Center, Karolinska Institutet, Stockholm, Sweden; e Division of Orthopaedic Surgery, McMaster University, Hamilton, Ontario, Canada

## Abstract

**Purpose:**

To evaluate the 2-year outcomes after arthroscopic surgical treatment for femoroacetabular impingement syndrome (FAIS) using validated patient-reported outcome measurements in young elite athletes and to report the rate of return to sport.

**Methods:**

Young elite athletes undergoing arthroscopic surgery for FAIS with 2 years of follow-up were included. A young elite athlete was defined as an athlete aged 18 to 22 years at the time of surgery with a Hip Sports Activity Scale (HSAS) level greater than 6 before the onset of symptoms. The following patient-reported outcome measurements were collected prospectively: Copenhagen Hip and Groin Outcome Score, 12-item International Hip Outcome Tool, HSAS, visual analog scale (VAS), European Quality of Life (EQ) 5 Dimensions questionnaire, and EQ VAS. Furthermore, the patients answered a question related to satisfaction with surgery at follow-up.

**Results:**

A total of 84 athletes (67 male and 17 female athletes), with a mean age of 19.8 ± 1.5 years, completed the 2-year follow-up. Bilateral hip arthroscopy was performed in 57 athletes, generating a total of 141 included hips. The improvements in the Copenhagen Hip and Groin Outcome Score subscales, 12-item International Hip Outcome Tool, EQ 5 Dimensions questionnaire, EQ VAS, and VAS for overall hip function were statistically significant (*P* < .001). At the 2-year follow-up, 42% of the athletes reported an HSAS level of either 7 or 8 whereas 28% reported an HSAS level of 5 or 6. In total, 79% of the athletes were satisfied with the surgical procedure.

**Conclusions:**

There are significant improvements in outcome measurements at the 2-year follow-up in elite young athletes undergoing arthroscopic hip surgery for FAIS. Although many of the athletes remained in high-level sports 2 years after surgery, only 30% of the athletes returned to sport at the same level.

**Level of Evidence:**

Level IV, therapeutic case series.

In recent years, femoroacetabular impingement syndrome (FAIS) has been recognized as a cause of hip pain in the athletic population.^1^^,^^2^ It has been suggested that FAIS impacts the biomechanics of the hip joint, leading to progressive hip pain and the early degeneration of the joint.^3^^,^^4^ The morphologic abnormalities of the femoral head (cam) and/or acetabulum (pincer) create the process of impingement in the hip joint, resulting in articular damage.^5^ It has been suggested that cam morphology in particular emerges during adolescence in response to high-impact sports, such as soccer and ice hockey.^4^ The diagnosis of FAIS is based on a combination of symptoms, clinical signs, and radiographic imaging findings.^6^ The arthroscopic treatment of FAIS has been the subject of widespread adoption in recent years.^7^^,^^8^ The goals of the treatment are primarily to relieve pain, by removing cam and pincer morphologies, and to address labral and/or cartilage injuries.

Prior studies have reported positive results in elite athletes undergoing arthroscopic hip surgery for FAIS.^9^^,^^10^ The results have mainly focused on returning to sport.^9^, ^10^, ^11^, ^12^ Studies have indicated signs of improvement after arthroscopic hip treatment in athletes, resulting in a high rate of return to preinjury activity levels, although there are limited measurements of returning to the same sport or performance level. Only a few studies have discussed the outcomes in terms of patient-reported outcome measurements (PROMs).^13^, ^14^, ^15^ Young elite athletes make up an important subgroup of patients undergoing arthroscopic hip surgery for FAIS. Although many studies have included this group of patients in the overall study group, the number of studies considering only this specific cohort is sparse.^14^ Several studies have considered adolescents; however, these patients have usually not completed their skeletal growth and have not yet reached the full potential of their athletic careers.^16^^,^^17^ On the other hand, previous data have indicated inferior outcomes with increased age,^18^ especially in terms of returning to sport^13^ and the risk of conversion to total hip arthroplasty in the general population.^19^ As a result, there is a risk of providing misleading results if studies include pooling of patients with a wide age range.^20^, ^21^, ^22^ Furthermore, evaluating the current clinical treatment in young elite athletes with a high rate of return to sports is of great importance.

The purposes of this study were to evaluate the 2-year outcomes after arthroscopic surgical treatment for FAIS using validated PROMs in young elite athletes and to report the rate of return to sport. We hypothesized that there would be improvements in clinical outcome scores and a high rate of return to sport among the elite athletes in this study.

## Methods

Patients registered in the Swedish hip arthroscopy registry^23^ and undergoing arthroscopic hip treatment for FAIS between January 2015 and December 2018 were evaluated for inclusion. The inclusion criteria included age between 18 and 22 years at the time of surgery and a Hip Sports Activity Scale (HSAS) level of 7 or 8 before the onset of symptoms. The exclusion criteria included patients missing any preoperative PROMs or patients who had undergone prior surgery on the hip. The FAIS diagnosis was based on a triad of clinical symptoms, radiologic findings, and physical examination findings according to the Warwick Agreement.^24^ The indication for hip arthroscopy was FAIS and failed nonsurgical treatment, whereas contraindications included severe forms of dysplasia or osteoarthritis.

For the arthroscopic treatment, a standard approach was used. The anterolateral and midanterior portals were established with the patient in a supine position on a traction table. The surgical technique has previously been described by Lindman et al.^13^ and Sansone et al.^25^ The patients were treated with the same step-by-step surgical procedure using traction, followed by stepwise resection of cam and pincer morphology; in addition, cartilage and labral injuries were addressed. Perioperative fluoroscopy was used during resection of bony abnormalities.

The athletes answered 1 set of PROMs for each surgical procedure, regardless of unilateral or simultaneous bilateral treatment. Other variables considered were the duration of symptoms, side of pain, FAIS morphology, classification of cartilage lesions according to the system of Konan et al.,^26^ duration of surgery, and duration of intraoperative traction. These variables were collected by the surgeon and completed perioperatively.

The HSAS is based on the Tegner Activity Scale^27^ and has been modified to better mirror different sports and the demands they impose on the hip joint. The HSAS consists of a scale from 0 to 8, where each increase in level reflects increased loads or physical demands on the hip joint. The lower end of the scale (level 0) indicates that the patient participates in no competitive or recreational sports, whereas the higher end (level 8) indicates participation in competitive sports at a national or international elite level.^28^^,^^29^

Web-based PROMs were answered by the athletes both preoperatively and at the 2-year follow-up. The athletes completed the Copenhagen Hip and Groin Outcome Score (HAGOS),^30^ 12-item International Hip Outcome Tool (iHOT-12),^31^ HSAS,^28^ visual analog scale (VAS) for overall hip function, European Quality of Life 5 Dimensions (EQ-5D) questionnaire,^32^ and European Quality of Life (EQ) VAS.^33^ The scores have previously been culturally validated for Swedish patients.^34^^,^^35^ In addition, at the 2-year follow-up, the athletes answered a question related to satisfaction with surgery (yes or no). The patients were all assessed by senior orthopaedic consultants.

In the rehabilitation protocol initiated after surgical treatment, patients were allowed free range of motion, as well as full weight bearing. The use of crutches was recommended for outdoor activities for 4 weeks after the operation. A standardized rehabilitation program focusing on strength, endurance, stability, coordination, and range of motion was implemented, the intensity of which was individualized to suit each patient in line with his or her symptoms. Patients received nonsteroidal anti-inflammatory drugs for 3 weeks after surgery to prevent heterotopic ossification.

### Statistical Methods

Demographic data are described with descriptive statistics. Continuous data are reported as median, range, mean, and standard deviation. Comparisons between PROM scores before surgery and at the 2-year follow-up were made using the Wilcoxon signed rank test. The level of significance was set at *P* < .05. The patient acceptable symptom state (PASS) threshold for the iHOT-12 score was set at 63.0 points, based on a previous study,^36^ whereas the minimal clinically important difference (MCID) for the iHOT-12 score and HAGOS was calculated as 0.5 times the standard deviation of the mean change in PROM scores.^13^

## Results

**Fig 1:**
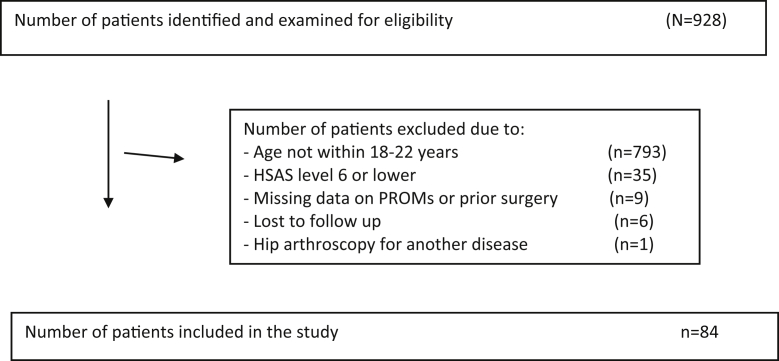
Flowchart of inclusion and exclusion criteria. (HSAS, Hip Sports Activity Scale; PROM, patient-reported outcome measurement.).

A total of 928 patients were evaluated for inclusion in the study (Fig 1); a total of 84 athletes completed the 2-year follow-up and were further included in the analysis (Table 1). The mean age was 19.8 ± 1.5 years, and there were 67 male participants (79.8%, 67 of 84). Bilateral hip arthroscopy was performed in 57 of the 84 athletes included (67.9%), generating a total of 141 included hips; in all cases, these procedures were performed simultaneously. The mean duration of symptoms before surgery was 30.9 ± 17.6 months.

**Table 1:** Demographic Characteristics

Characteristic	Data
Patients, n	84
Hips, n	141
Female/male, n (%)	17 (20.2)/67 (79.8)
Age, yr	19.8 (1.5)
Bilateral/unilateral surgery, n (%)	96 (68)/45 (32)
Operated side: right/left, n (%)	70 (49.6)/71 (50.4)
Symptom duration, mo	30.9 (17.6)
Operation time, min	56.8 (14.8)
Traction time, min	4.7 (2.7)
Konan classification, %	
0	18
1a	11
1b	2
1c	0
2	22
3a	24
3b	6
3c	0
4a	1
4b	2
4c	0
Not visualized	8
Missing data	6

NOTE. Data are presented as mean (standard deviation) unless otherwise indicated.

SD, standard deviation.

Combined cam and pincer morphology was found in 110 of 141 hips (78%), whereas isolated cam morphology was found in 30 (21%) and isolated pincer morphology was only found in 1 (1%). Other procedures performed during surgery were microfracture, labral debridement, labral suture, labral resection, and ligamentum teres resection (Table 2).

**Table 2:** Surgical Procedures (141 Hips)

Surgical Procedure	Hips, n (%)
Cam treatment	30 (21)
Pincer treatment	1 (1)
Combined treatment (treatment of both cam and pincer morphology)	110 (78)
Labral suture	1 (1)
Labral debridement	3 (2)
Labral resection	1 (1)
Microfracture	5 (4)
Ligamentum teres resection	1 (1)

The mean improvements in the HAGOS subscales, iHOT-12 score, EQ-5D score, EQ VAS score, and VAS score for overall hip function from preoperatively to the 2-year follow-up were all statistically significant and clinically relevant (Table 3). At the 2-year follow-up, approximately 79% of the athletes were satisfied with the surgical procedure (Table 3). For the HAGOS subscales, the MCID values were exceeded by 77% of athletes for symptoms, 74% for pain, 50% for function in daily activity, 70% for sports, 64% for physical activity, and 68% for quality of life. Approximately 2 in every 3 athletes exceeded the PASS (67%) and the MCID (65%) for the iHOT-12.

**Table 3:** PROMs and MCIDs

PROM	Preoperative	24 mo	Change	*P* Value	MCID
HAGOS					
Symptoms	50.8 (18.2)	71.4 (19.6)	20.6 (20.4)	<.001	10.2
Pain	58.3 (18.0)	80.3 (19.0)	22.1 (19.6)	<.001	9.8
Daily activity	63.2 (23.1)	81.5 (23.9)	18.3 (25.0)	<.001	12.5
Sports	39.7 (21.4)	70.8 (25.8)	31.1 (25.5)	<.001	12.8
Physical activity	31.0 (24.9)	65.6 (34.6)	34.7 (36.6)	<.001	18.3
Quality of life	30.7 (18.9)	63.8 (29.2)	33.1 (27.2)	<.001	13.6
iHOT-12 score	45.1 (16.8)	70.8 (26.3)	25.7 (24.0)	<.001	12.0
EQ-5D score	0.6 (0.3)	0.8 (0.3)	0.2 (0.3)	<.001	NA
EQ VAS score	65.0 (18.4)	77.0 (16.7)	12.0 (20.1)	<.001	NA
VAS score for overall hip function	49.3 (20.8)	72.0 (22.4)	22.7 (23.4)	<.001	NA
HSAS level	7.5 (0.5)	5.4 (2.0)	–2.1 (2.0)	<.001	NA
Satisfied with surgery, %	NA	78.6	NA	NA	NA

NOTE. Data are presented as mean (standard deviation) unless otherwise indicated.

EQ, European Quality of Life; EQ-5D, European Quality of Life 5 Dimensions questionnaire; HAGOS, Copenhagen Hip and Groin Outcome Score; HSAS, Hip Sports Activity Scale; iHOT-12, 12-item International Hip Outcome Tool; MCID, minimal clinically important difference; NA, not applicable; PROM, patient-reported outcome measurement; VAS, visual analog scale.

Approximately half of the young elite athletes (51%, 43 of 84) reported an HSAS level of 8 before the onset of symptoms (Table 4). At the 2-year follow-up, 42% of the athletes (35 of 84) reported an HSAS level of either 7 or 8 whereas 28% (23 of 84) reported an HSAS level of either 5 or 6. Thirty percent of the athletes were performing at the same HSAS level or at a higher HSAS level at the 2-year follow-up compared with before the onset of symptoms. Furthermore, 11% of the athletes (9 of 84) were still participating in sports at an elite level according to the HSAS—at level 7—despite reporting a decrease in the HSAS level from 8 to 7, whereas 1 athlete showed an increase in the HSAS level from level 7 before the onset of symptoms to level 8 at the 2-year follow-up. A total of 69% of the athletes (58 of 84) were still found to have an HSAS level of 5 to 8 at the 2-year follow-up.

**Table 4:** HSAS Levels

HSAS Level	Before Symptoms	At Time of Surgery	2-yr Follow-up
0		6 (5 of 84)	2 (2 of 84)
1		15 (13 of 84)	1 (1 of 84)
2		6 (5 of 84)	2 (2 of 84)
3		15 (13 of 84)	13 (11 of 84)
4		6 (5 of 84)	12 (10 of 84)
5		10 (8 of 84)	24 (20 of 84)
6		5 (4 of 84)	4 (3 of 84)
7	49 (41 of 84)	13 (11 of 84)	23 (19 of 84)
8	51 (43 of 84)	24 (20 of 84)	19 (16 of 84)

NOTE. Data are presented as percentage (number).

HSAS, Hip Sports Activity Scale.

At the 2-year follow-up, 79% of the athletes (66 of 84) reported that they were satisfied with the surgical procedure. There were no complications.

## Discussion

The main finding in this study was that young elite athletes who underwent hip arthroscopy for FAIS showed marked improvements in PROMs at the 2-year follow-up, although only 42% of all athletes were able to resume their preinjury level of activity. The young elite athletes reported significant improvements in all PROMs except the HSAS. Most of the athletes reached the MCID for the iHOT-12 and HAGOS subscales, as well as the PASS for the iHOT-12, indicating a clinically relevant improvement in hip function. At the 2-year follow-up, 42% of the athletes reported an HSAS level of either 7 or 8 whereas 28% reported an HSAS level of 5 or 6. In total, 79% of the athletes were satisfied with the surgical procedure.

Studies have previously reported favorable PROMs for athletes after undergoing hip arthroscopy for FAIS,^13^^,^^37^^,^^38^ although the vast majority of previous studies have included patients with a wide range of ages or have focused on adolescents. In this study, we aimed to narrow the age range to obtain a more homogeneous group of patients to evaluate the outcomes in patients at an important time in their elite careers. The results of this study are in line with the findings of previous studies of athletes undergoing hip arthroscopy for FAIS,^13^^,^^39^^,^^40^ indicating a significant functional improvement, even though fewer than half of the patients can expect to return to pre-symptom levels. In recent years, efforts to define improvements in outcomes for patients have been increasing.^41^ These outcomes include the MCID and PASS and should surely be the benchmark for treatment evaluation. However, it is important to acknowledge the results for the HAGOS and iHOT-12 score. The HAGOS results revealed that most patients exceeded the MCID values for all subscales, although the assessments of function in daily activity and physical activity yielded the lowest values. Only 50% of the patients exceeded the MCID value for function in daily activity, but it has previously been reported that this subscale has a risk of a ceiling effect.^42^ With a mean value at follow-up of 81.5, compared with a baseline value of 63.2, it will be important to continue to delineate the specific functional limitations that exist, despite the overall improvement. Furthermore, only 64% of the patients exceeded the MCID value for physical activity. Although the patients improve after hip arthroscopy, the possibility that they may not recover fully in terms of physical activity has previously been discussed.^43^^,^^44^ These values may also reflect the high expectations of the athletes in our study. Previous registry results indicated a range of 63% to 73% of patients who exceeded the MCID at the 1-year follow-up,^45^ which is in line with the results of our study.

The iHOT-12 results are furthermore indicative of acceptable results after surgery, with 2 in every 3 patients experiencing an improvement in terms of exceeding the MCID value. These results are consistent with those of a recent study by Beck et al.^46^ reporting positive outcomes for the adolescent patient after hip arthroscopy for FAIS. Furthermore, the International Hip Outcome Tool is a valuable tool for identifying substantial clinical benefit after hip arthroscopy for FAIS in young active patients.^47^ In a recent study by Ishøi et al.,^42^ attempts were made to measure how many patients achieved the PASS after surgical treatment of FAIS. There were no measurements of the patients’ activity levels. In the previous study, 46% of the patients achieved the PASS at follow-up performed 12 to 24 months after surgery compared with 67% of the patients in our study. It would be favorable to be able to compare the current study to Ishöj et al, but the age difference is a limiting factor since the average age of the patients included in the study from Ishöj et al, was considerably older than in the current study (35 years versus 20 years). It is important to consider that, although the MCID values reveal a significant improvement, there is limited information on patient satisfaction.

Despite a mean decrease in the HSAS level in this study, 42% of the patients remained active as elite athletes at an HSAS level of 7 or 8. Furthermore, at the 2-year follow-up, 30% of the athletes performed at the same HSAS level as that prior to the onset of symptoms. While bearing in mind the fact that a further 28 patients remained at an HSAS level of 5 or 6 (a total of 70% of patients at level 5-8), our results are compatible with those of Lindman et al.,^13^ who reported that 54% of patients had HSAS levels of 5 to 8 at 5-year follow-up. The patients in our study had an average age of 19.8 years, whereas those in the study of Lindman et al. had an average age of 24.6 years. It is noteworthy that the follow-up in our study was completed at 2 years, whereas Lindman et al. conducted a 5-year follow-up of their patients. The results in our study indicate that, at a mean age of almost 20 years, 70% of the patients were still performing at HSAS levels of 5 to 8 at 2 years after surgery.

Because the HSAS is not designed to measure actual return to sport, the conclusions that can be drawn from this result are questionable. Litrenta et al.^16^ examined adolescent athletes treated with hip arthroscopy for FAIS. Their study only included patients attempting to return to sport and showed that 84% reported a return to sport, with 77% reporting that their competitive ability was at a level similar to or higher than 1 year before the surgical procedure. However, most of the patients underwent labral repairs and fractional iliopsoas lengthening, whereas in our study, osseous pathology was addressed, with only a few cases of other surgical procedures. Return to sport is also defined without any attempt to evaluate the level of intensity or the patient’s own expectations or wish to return to sport, and many factors may impact this decision.^48^ In a study of patients in the Danish Hip Arthroscopy Registry, the percentage of young athletes returning to their preinjury level was 57% after undergoing hip arthroscopy for FAIS.^15^ However, no strict definition of “athlete” was used in comparison with our study, which had more strict criteria when applying the HSAS. Return-to-sport decisions are often complex and seldom reflect surgical intervention alone.

Even though this study points toward an overall improvement after hip arthroscopy, our results show that a few patients do not experience a full recovery and are unable to return to their level of activity according to the HSAS after surgical treatment. It is noteworthy that the HSAS level was evaluated before the onset of symptoms, with a mean duration of symptoms before treatment of 30.9 months. It has been noted by Lindman et al.^13^ that patients with a long symptom duration prior to surgery generally report lower HSAS levels at follow-up assessments. It is, however, a challenge to measure return to sport in this study because the relevant PROMs do not gauge the individual patients’ desired activity level. This is particularly pertinent when considering return to sport because studies have shown that physical activity in general among adolescents decreases with higher age,^49^ even without debilitating injuries.

The prevalence of bilateral hip arthroscopy for FAIS in this study is high compared with the findings of Klingenstein et al.,^50^ who reported bilateral treatment in only around 20% of patients. In their study, the identified risk factors for bilateral symptoms included young age, male sex, and bilateral radiographic findings. This could explain why the patients in our study—predominantly young male athletes—were often treated bilaterally. Previous findings reported by McConkey et al.^51^ showed no difference in outcome measurements in patients undergoing hip arthroscopy for FAIS unilaterally compared with those undergoing bilateral treatment.

The strengths of this study include the prospective collection of data, the well-defined inclusion criteria for young elite athletes, and the validated PROMs for a young and active population. In this study, efforts have been made to perform state-of-the-art patient evaluations.^52^

### Limitations

One major limitation of this study is the lack of a control group. Furthermore, the HSAS scale is not primarily designed to measure return to sport, and no consideration has been taken of the patients’ ambition to return to sport. However, the literature is noted to have varying definitions of “return to sport,” which this study addressed by using the HSAS.^53^ These young elite athletes were included according to radiographic findings in line with the Warwick Agreement,^24^ although the lack of radiographic imaging after the operation limits the ability to evaluate the disease progression radiographically.

## Conclusions

There are significant improvements in outcome measurements at the 2-year follow-up in elite young athletes undergoing arthroscopic hip surgery for FAIS. Although many of the athletes remained in high-level sports 2 years after surgery, only 30% of the athletes returned to sport at the same level.

## Supplementary Data

ICMJE author disclosure forms
